# The effect of combined oral contraceptives on thrombin generation assessed on ST Genesia– a paired clinical study

**DOI:** 10.1186/s12959-025-00713-z

**Published:** 2025-04-08

**Authors:** Jesper Strandberg, Inger Lise Gade, Jette Nybo, Janus Nikolaj Laust Thomsen, Søren Risom Kristensen

**Affiliations:** 1 The Coagulation Unit, Department of Clinical Biochemistry, Aalborg University Hospital, Aalborg, Denmark; 2 Department of Clinical Medicine, Aalborg University, Aalborg, 9000 Denmark; 3 Department of Haematology and Clinical Cancer Research Centre, Aalborg University Hospital, Aalborg, Denmark; 4 Center for general practice, Aalborg University, Aalborg, Denmark

**Keywords:** Thrombogenicity, Combined oral contraceptives, Thrombin generation, Coagulation, Venous thromboembolism

## Abstract

**Background:**

Treatment with combined oral contraceptives (COC) is associated with an increased risk of venous thromboembolism. Several changes of coagulant and anticoagulant factors induced by ethinyloestradiol during treatment with COC, have been demonstrated. Thrombin generation is a global test measuring both coagulant and anticoagulant factors, but the effect of COC on individuals starting COC, has not been examined before on the new equipment, ST Genesia. The aim of this project was to examine the effect of COC on thrombin generation on ST Genesia, in individuals before and after starting COC.

**Methods:**

Twenty-four female participants between 15 and 34 years of age, who were about to start treatment with ethinylestradiol/levonorgestrel-containing COC, were included in the study. Two blood samples were drawn from each of the study subjects, a baseline sample immediately before first COC dose, and a follow-up blood sample approximately 3–4 months after COC start. Standard biochemical analyses as well as standard and special coagulation analyses including thrombin generation on ST Genesia, were performed in all samples.

**Results:**

Thrombin generation, i.e., endogenous thrombin generation (ETP) and peak increased considerably after COC start, whereas time-to-peak was shortened. Thrombin-antithrombin complexes (TAT), prothrombin fragments (F1 + 2) and sex hormone binding globulin (SHBG) increased, and the coagulation inhibitors tissue factor pathway inhibitor (TFPI), protein S activity and antithrombin decreased slightly after COC start.

**Conclusion:**

Although the coagulation factors only changed modestly, the global test thrombin generation performed on ST Genesia showed a considerable change after start of COC.

**Supplementary Information:**

The online version contains supplementary material available at 10.1186/s12959-025-00713-z.

## Introduction

Combined oral contraceptives (COC) is a common method of anticonception worldwide with an estimated number of users of about 150 million women [1]. Most COC contain an artificial oestrogen component, ethinyloestradiol (EE), and a progestogen; the type of progestogen has been classified COC into four generations, but COC may also be stratified based on its contents, i.e. dose of EE and the type of progestogen [2–4]. The 2nd generation COC contain 20–30 µg EE combined with levonorgestrel, 100–150 µg, respectively. The higher dose 150 µg of levonorgestrel helps to counterbalance the higher oestrogen effect of 30 µg EE. A few types contain 35 µg EE and 250 µg norgestimate. In 3rd generation COC, the EE dose also varies between 20 µg and 30 µg, but contain other types of progestogens, desogestrel (150 µg) or gestodene (75 µg). These progestogens have a lower counterbalancing effect on EE, leading to greater impact on the coagulation system and a higher VTE risk profile for 3rd generation COC [2, 4, 5]. The 4th generation COC contain the progestogen drospirenone, but is much like 3rd generation COC. Besides, some types of COC are unclassified including a type containing 35 µg EE and 2 mg cyproterone acetate. Recently, a new COC formulation with estetrol, a natural oestrogen, has been introduced, which is thought to have a lower impact on the coagulation system, and therefore a potentially lower risk of VTE [4, 6–9]. In general, however, treatment with COC is associated with an increased risk of venous thromboembolism (VTE) [6, 10, 11]. 

VTE is a worldwide public health problem and occurs at an annual incidence of between 100 and 200 per 100.000 adults [12–14]. The incidence is higher for COC users than non-users and is age dependent [15, 16]. According to Lidegaard et al. [15] the incidence for VTE among COC users is 42 per 100.000 women years for 15-19-year-olds, increasing to 208 per 100.000 women years for 45-49-year-old, being higher for users of third and fourth generation than second generation COC. Corresponding figures for non-users are 7 (15–19 years old) increasing to 58 (45–49 years old) per 100.000 women years.

Oestrogen causes prothrombotic alterations in proteins involved in the coagulation process, and use of COC leads to increased levels of some procoagulant factors and decreased levels of anticoagulant factors [4, 17–20]. However, most of these changes are modest, but the global assay thrombin generation including all procoagulant and anticoagulant factors can demonstrate an increased thrombin generation after initiation of COC, indicating a hypercoagulable state and a potentially higher risk of VTE [21–27]. The thrombin generation test can be extended by adding thrombomodulin (TM) activating Protein C, which includes the effect of the Protein C/Protein S system in the thrombin generation test. Kristensen et al. [28] measured thrombin generation on a new coagulation platform, ST Genesia, and demonstrated a considerably higher thrombin generation in COC users compared with non-users. ST Genesia is a rather new equipment meant as a fully automated routine instrument in coagulation labs [28] where the CAT instrument mainly is used as a research tool. This new platform includes specialized kits, of which ST Thromboscreen can be used to investigate potential thrombophilia. Measurement of thrombin generation including thrombomodulin in women starting treatment with COC has to our knowledge only been done by Zia et al. [29] in 2015 in a group of 33 women, and by Westhoff et al. [24] in 16 women during the first cycle after start of COC, both measuring thrombin generation on the CAT system.

The main objective of this project was to evaluate the intraindividual effect of COC on thrombin generation analysed on ST Genesia, as a tool to demonstrate the intraindividual variations. This might be used as a reference to demonstrate unusual thrombogenic effects of COC in thrombosis prone individuals and can potentially have impact on the counselling of women regarding the start-up of COC in the future.

## Methods

### Study design and study population

In several larger medical practices in the North Denmark Region, healthy women between 15 and 50 years of age, who were about to start treatment with COC, were invited to participate. Exclusion criteria were a history of arterial or venous thrombosis, anticoagulant treatment, underlying intercurrent diseases (e.g., PCOS, diabetes, inflammatory bowel disease etc.), and recent hospitalization or surgery. Subjects who previously used COC or progesterone-only pills were invited to participate if they had a minimum of two months oral contraception-free period before the baseline visit/blood sample. In Denmark, the use of a 2nd generation COC is recommended as the primary choice, but the decision is made at the discretion of the local physician.

A total of 30 women were included in the study between 2nd of June 2021 and 27th of September 2023. Four participants dropped out of the study prematurely, three due to stopping COC before the follow-up visit and one due to loss of contact; those participants were therefore excluded from the study.

The subjects were asked to donate a baseline blood sample before starting COC use, and a follow-up blood sample approximately 3 menstrual cycles after initiation of the treatment with COC. On the day of the baseline visit, the subjects filled out a questionnaire, concerning age, smoking, BMI, family history of VTE, history of surgery, underlying diseases, earlier and present medication. Blood samples and questionnaires were collected at the Department of Biochemistry at Aalborg University Hospital, Denmark. To ensure the inclusion of potential participants, we did not impose any specific requirement regarding the phase of the menstrual cycle for the baseline blood sample, and therefore the first blood samples were taken at different times during the menstrual cycle, although mainly around the menstruation. The follow-up blood sample was attempted to be taken on days 10–14 of their menstrual cycle.

The study was approved by the regional Danish research ethics committee (N-20200098 ) and registered at Aalborg University Hospital. Informed written consent was obtained for all subjects. For participants under 18 years, written consent from both parents were also obtained.

### Blood collection

Venous blood was collected from the antecubital fossa using a 21-gauge needle into EDTA-, LiHep- and 3.2% sodium citrate vacutainers for the standard biochemical analyzes, and into 3.2% (w/v) trisodium citrate Monovette tubes (Sarstedt, Nümbrecht, Germany) for the thrombin generation analyzes. The first tube was discarded. The Monovette samples were centrifuged twice at 2500 G for 15 min at 20 °C immediately after collection in accordance with current guidelines [30]. Plasma samples were stored at -80 °C until analysis.

### Biochemical investigation

In plasma (P-) and whole blood (B-), standard clinical biochemical analyses (P-Kalium, P-Sodium, P-Albumin, P-Carbamide, P-Creatinine, P-Alanine transaminase(ALAT), P-Lactate dehydrogenase (LDH), P-Bilirubin, P-C-reactive protein (CRP), P-Cholesterol, P-High Density Lipoprotein (HDL), P-Low Density Lipoprotein (LDL), P-Triglycerides) and haematology analyses (B-Haemoglobin, B-Erythrocytes, B-Erythrocyte volume, mean (MCV), B-Erythrocyte volume fraction (EVF), B-Reticulocytes, B-Leukocytes, B-Thrombocytes and B-Thrombocyte volume, mean (MPV), B-Immature Platelet Count (IPC) and B-Immature Platelet Fraction (IPF)) were performed on Abbott Alinity and Sysmex XN-9100, respectively, using dedicated reagents.

Coagulation analyses included P-Prothrombin time/INR, P-Activated Partial Thromboplastin Time (aPTT), P-D-dimer, P-Fibrinogen, functional protein C, free protein S antigen concentration and protein S activity, antithrombin, measured with both anti-IIa (AT-T)- and anti-Xa-method (AT) ((all performed on ACL TOP500), as well as genetic polymorphisms of coagulation factors II and V. Sex hormone binding globulin (SHBG) levels were measured on Cobas 6000, module 601E (Roche). Thrombin-Antithrombin Complexes (TAT) and prothrombin fragments (F1 + 2) were determined by ELISA methods (Enzygnost F1 + 2 (monoclonal), Siemens Healthcare, REF: OPBD03; Enzygnost TAT micro, Siemens Healthcare, REF: OWMG15) as well as tissue factor pathway inhibitor (TFPI) (Asserachrom, Stago).

The thrombin generation test providing an overall assessment of the coagulation system, including all the effects of coagulation and anticoagulation factors was measured on ST Genesia (Diagnostica Stago, France), with (+ TM) and without (-TM) thrombomodulin using the reagent ST Thromboscreen and performed as described before [28]. Information of the concentrations of tissue factor and phospholipids are not available, but the reagent is intended to be used for patients with possible thrombophilia. The coagulation is activated by addition of tissue factor and the formation of thrombin generation is followed after addition of a fluorophore (Z-Gly-Gly-Arg-AMC) which is cleaved by thrombin indicating the thrombin activation. The results are described as the lag time which is the time until the first formation of thrombin, peak height which is the maximal formation of thrombin, time-to-peak which is the time from start to the peak of thrombin generation, and endogenous thrombin potential, which is the total formation of thrombin (the area under the curve (a thrombogram is illustrated in supplementary Fig. 1). An ETP inhibition ratio, an expression of the impact of TM on ETP, is defined as: 100 ((ETP_without TM_ - ETP_with TM_)/ETP_without TM_).

### Statistical considerations

The aim of the study was initially to establish reference intervals for ST Genesia. We planned to include 60–80 participants, which we anticipated to be a possible achievement, slightly less than the 120 participants prescribed by guidelines [31, 32], but giving a reasonable estimate. However, the recruitment was astonishingly slow, and consequently the number of participants ended on 26 participants. Therefore, we only describe the results as an indication of reference intervals.

Wilcoxon signed-rank test was used to test the statistical differences between the baseline and follow-up results, and the results are presented as median, and 25-, and 75-percentiles. All differences were also tested parametrically (paired Student’s t-test), showing virtually the same results. Data distributions were assessed by histograms and Quantile-Quantile plots. Student’s t-test was used in normally distributed data, and in case the assumption of normal distribution was not fulfilled estimates were based on the Wilcoxon signed-rank, and differences were considered significant when the P-value was < 0.05. The associations between different analytes were assessed using Pearson’s correlation coefficients and are described as R^2^.

## Results

### Basic characteristics of the study population

A total of 26 subjects were included in the study, with a median age of 17 years at the day of the first blood sample. Six of the study participants used medicine for either attention deficit hyperactivity disorder (ADHD), acne, migraine, heart palpitations or allergy. Six of the subjects had previously used COC, but not within the last 3 months prior to inclusion, and five subjects used regular vitamin pills. None of the subjects had a family history of VTE. None of the subjects had either a PCOS diagnosis or menstrual cycle irregularities. None of the subjects were former or active smokers. Almost all subjects, 24 women (92%), received 2nd generation COC, of which four subjects (17%) received 20 µg EE and 100 µg levonorgestrel and the rest (83%) received 30 µg EE and 150 µg levonorgestrel. One subject received 3rd generation COC (30 µg of EE and 75 µg of gestodene) and one subject received a generation-unspecified COC, containing 2 mg cyproterone acetate and 35 µg EE. Due to this, we decided not to include these subjects in further analysis or comparisons. Study population characteristics are presented in Table 1.

**Table 1:** Basic characteristics of study population

Subjects	26
Age, median (range) (years)	17 (15–34)
BMI (baseline), median (range) (kg/m^2^)	21.6 (17.9–28.6)
BMI (follow-up), median (range) (kg/m^2^)	22.3 (18,3–29.5)
Active/Former smoker	None/None
Family history of VTE	None
Ethinyloestradiol dose (20 µg/30µg/35µg)	4/21/1
COC generation, 2nd /3rd	24/1^A^
Day in menstrual cycle for follow-up blood sample, median (range)	12 (9–19)

A = One subject received a generation-unclassified COC containing 35 µg of ethinyloestradiol and 2 mg of cyproterone acetate. BMI = Body Mass Index, COC = Combined oral contraceptives

Eleven (46%) of the baseline blood samples were taken right before menses, five (21%) during, and three (12%) right after menses. The remaining five (21%) were taken approximately two weeks after the last day of menses.

### Biochemical measurements

The results for the standard biochemical analyses were all within the normal ranges and are presented in supplementary Table 1. No statistically significant differences between the baseline blood sample and the follow-up blood sample were observed, and all values were within the normal reference intervals. For CRP, there was a small, but not statistically significant, increase from baseline to follow-up.

### Coagulation related measurements

SHBG as a marker of the oestrogen effect increased markedly, i.e., 87% (*p* < 0.0001) but for the coagulation related measurements the changes were minor (Table 2). Antithrombin decreased slightly with the anti-IIa method whereas it surprisingly was unchanged with the anti-Xa method. Protein S activity also decreased, whereas free protein S antigen did not change. TFPI decreased significantly and TAT and F1 + 2 increased.

**Table 2:** Results for coagulation analyses, *n* = 24

Analyte, unit	Baseline, median Median [25p-75p]	Follow-up (median) Median [25p-75p]	Absolute intraindividual difference Mean ± SD	Relative intraindividual difference Mean ± SD	*p*-value
INR	1.1 [1.0–1.1]	1.1 [1.0–1.1]	-0.008 ± 0.08	-0.7 ± 6.9	0.641
aPTT, s	27 [25–28]	27 [24–29]	-0.29 ± 3.85	-0.3 ± 13.7	0.687
D-dimer, mg/L FEU	0.31 [0.09–0.42]	0.28 [0.0–0.42]	− 0.03 ± 0.31	-18 ± 75	0.674
Fibrinogen, µmol/L	8.0 [7.1–8.7]	7.6 [6.1–9.6]	-0.3 ± 2.1	-2 ± 24	0.623
SHBG, nmol/L	47 [38–61]	88 [68–109]	38 ± 26	82 ± 60	< 0.0001
TFPI, ng/mL	44 [40–47]	39.4 [37–42]	− 4.7 ± 5.4	-10 ± 11	0.0002
AT-T	1.08 [1.04–1.14]	0.99 [0.92–1.05]	-0.08 ± 0.06	-8 ± 6	< 0.0001
AT, x10^3^ IU/L	0.98 [0.86–1.09]	0.98 [0.92–1.05]	0.001 ± 0.15	2 ± 16	0.964
Functional protein C, arb. u/L	1.04 [0.95–1.13]	1.05 [0.96–1.17]	0.02 ± 0.15	2 ± 15	0.566
Free protein S	0.95 [0.85–1.08]	1.08 [0.85–1.13]	0.04 ± 0.17	5 ± 18	0.247
Protein S activity	1.00 [0.91–1.07]	0.93 [0.88–1.01]	-0.05 ± 0.10	-5 ± 10	0.025
TAT, µg/L	2.5 [2.2–2.6]	2.6 [2.3–3.3]	0.4 ± 0.6	15 ± 23	0.004
F1 + 2, pmol/L	109 [86–144]	137 [115–183]	33 ± 43	35 ± 43	0.001

INR = international normalized ratio. aPTT = activated partial thromboplastin time. AT = antithrombin activity measured by anti-Xa-method. AT-T = antithrombin activity measured by anti-IIa-method. TFPI = tissue factor pathway inhibitor. SHBG = sexual hormone binding protein. TAT = thrombin-antithrombin complex. F1 + 2 = prothrombin fragments 1 + 2

Two subjects were heterozygous for factor V Leiden, and one was heterozygous for factor II of prothrombin gene G20210A.

### Thrombin generation

**Fig. 1:**
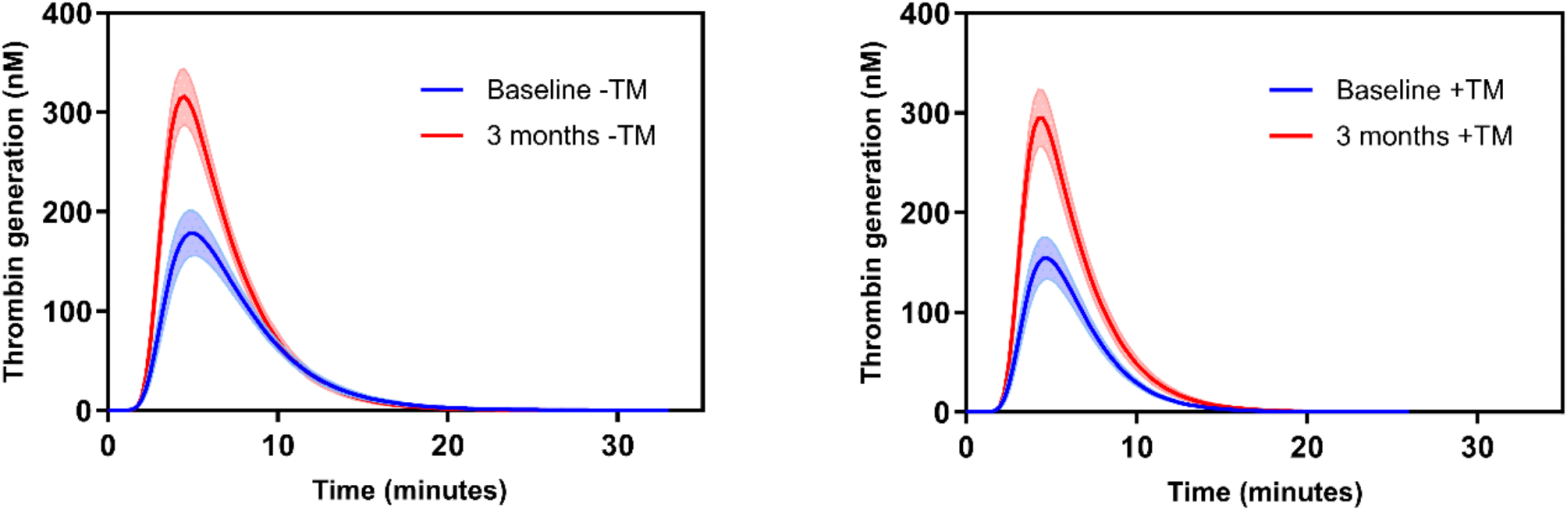
Baseline and 3-months follow-up thrombin generation results, presented as mean values with 95% confidence intervals (CI), with (+ TM) and without (-TM) thrombomodulin. TM = thrombomodulin

The results for thrombin generation are presented in Table 3, with statistically significant changes for most of the values: A decreased time-to-peak in combination with a rather large increase of ETP, velocity index and peak height at the follow-up. In the presence of TM was seen similar changes, but the increases of peak height and ETP at the follow-up were relatively higher. The differences in thrombin generation, with and without thrombomodulin, is illustrated in Fig. 1.

**Table 3:** Results for thrombin generation associated analyses. *n* = 24

Thrombin generation	Baseline Median [25p-75p] 95% CI	Follow-up Median [25p-75p] 95% CI	Absolute individual difference Mean ± SD	Relative individual difference, Mean ± SD (%)	*p*-value
TG (-TM), unit					
Lag time, min	2.4 [2.3–2.6] 2.1–3.1	2.4 [2.3–2.5] 2.2–2.8	-0.05 ± 0.2	-1 ± 8	0.705
Peak height, nmol/L	172 [134–227] 107–268	319 [277–363] 182–445	139 ± 65	88 ± 54	< 0.0001
Time-to-peak, min	5.1 [4.8–5.4] 4.5–6.0	4.5 [4.3–4.7] 4.1–5.0	-0.6 ± 0.4	-11 ± 6	< 0.0001
ETP, nmol/L.min	1167 [1010–1274] 867–1570	1657 [1522–1812] 1220–2191	511 ± 222	46 ± 24	< 0.0001
Velocity Index, nM/min	85 [67–130] 50–152	206 [171–243] 106–310	113 ± 50	137 ± 85	< 0.0001
TG (+ TM), unit					
Lag time, min	2.5 [2.4–2.7] 2.2–3.3	2.5 [2.4–2.6] 2.3–2.9	-0.04 ± 0.2	-1 ± 8	0.877
Peak height, nmol/L	153 [118–203] 79–224	292 [260–350] 161–428	142 ± 66	105 ± 67	< 0.0001
Time-to-peak, min	4.7 [4.5–4.9] 4.3–5.6	4.4 [4.2–4.6] 4.1–4.8	-0.4 ± -0.3	-7 ± 5	< 0.0001
ETP, nmol/L.min	792 [610–969] 406–1136	1384 [1177–1592] 816–1930	600 ± 287	88 ± 55	< 0.0001
Velocity Index, nM/min	95 [65–131] 48–149	213 [176–253] 105–315	117 ± 53	144 ± 91	< 0.0001
ETP inhibition ratio	34 [25–43] 20–53	18 [14–23] 8–32	-16 ± 11	-43 ± 30	< 0.0001

95%CI = The 95% confidence interval with 2.5th and 97.5th percentiles. TG = thrombin generation. TM = thrombomodulin. ETP = endogenous thrombin generation. ETP inhibition ratio = 100 ((ETP_without TM_ - ETP_with TM_)/ETP_without TM_). TM = thrombomodulin

The ETP inhibition ratio is an indication of the effect of the protein C: protein S system. We found lower ETP inhibition ratio in the follow-up samples compared to baseline of approximately 50%; (*p* < 0.0001). The two subjects heterozygous of factor V Leiden showed reduced ETP inhibition ratios of 57% and 7% respectively.

We investigated the correlations between increased thrombin generation and changes in some analytes. There was a moderate correlation between an increase of ETP and an increase of SHBG (R^2^ = 0.24), indicating that the effects on coagulation correlates to oestrogenicity. We observed moderate correlations between increased thrombin generation and decreased levels of TFPI and AT-T, and those tendencies applied to both higher ETPs (R^2^ values of 0.45 and 0.31 respectively) and peak values (0.46 and 0.26 respectively), as well as shorter time-to-peak (0.27 and 0.15 respectively). The correlation between thrombin generation and TFPI and AT-T, indicates that a substantial effect on thrombin generation is caused by the reduction of coagulation inhibitors. Thrombin generation also correlated to the relatively small increase of F1 + 2, i.e., the coagulation activity. No correlation was seen for thrombin generation and protein S activity. Correlations are illustrated in supplementary Fig. 2. The two excluded women who did not receive 2nd generation COC had values for thrombin generation within the intervals for the other women.

Although the 20 µg-group was small (4 participants), (low statistical power) we compared them with the 30 µg- group. Using parametric statistics, we compared the relative difference values between the groups, and the 30 µg-group showed a remarkably higher, statistically significant increase in SHBG, F1 + 2 and TAT, but no significant changes in ETP, than the 20µ-group. Comparisons are presented in supplementary Table 2.

The subject who was heterozygous for the prothrombin gene G20210A had the second greatest increase in ETP (89%), whereas the two subjects with Factor V Leiden had thrombin generation increases of approximately 15% and 43%, which were in the lower end and in the middle of the distribution, respectively.

## Discussion

Our study demonstrates that thrombin generation, assessed on ST Genesia, shows a clear and significant thrombogenic effect of COC. This was supported by slightly but significantly increased levels of TAT and F1 + 2. Tissue factor pathway inhibitor, AT-T and protein S activity decreased significantly, but the changes were only modest whereas the effect on TG was substantial, which shows that the combined effects of all changes were considerable. The increased levels of SHBG indicate that this is an oestrogenic effect.

The thrombin generation measures the global coagulation system including all the coagulation factors and the effects of TFPI and antithrombin. Although some coagulation factors increase after start of COC it does not cause significant changes of aPTT and INR, and the change of TG is probably mainly caused by changes of the coagulation inhibitors, especially TFPI [33]. Addition of thrombomodulin will also measure the effect of Protein S in combination with activated Protein C, as thrombomodulin forms a complex with thrombin activating protein C. Therefore, the thrombin generation is even more enhanced (especially Peak and ETP) in the presence of thrombomodulin (ETP increases 75% (+ TM) vs. 42% (-TM) at follow-up compared with baseline), even though the Protein S activity was only moderately reduced, and, consequently, the ETP inhibition ratio is also reduced to approximately the half of baseline after start of COC. Thrombin generation in users of COC has been measured with the CAT method in several studies, but results measured on ST Genesia has not been reported before. ST Genesia is a routine instrument, and it will potentially be used in routine coagulation labs in the future. Therefore, the effect of COC using this instrument can be an important measurement in order to consider thrombogenicity in COC users. The reagent ST Thromboscreen is meant for determination of thrombogenicity, and the present results is an indication of expected changes after start of COC use.

A general increase of thrombin generation associated with COC is well documented in the literature [23, 25, 34–36]. Kristensen et al. [28] showed that thrombin generation measured on ST Genesia was considerably higher in COC users than non-users. Our results are in line with this previous study, but here we assessed thrombin generation performed on the same subjects before and after start of COC giving a better estimate of the individual changes. We did not measure APC sensitivity ratio, which is not possible on ST Genesia, but the ETP inhibition ratio is a comparable indication of reduced sensitivity of activated protein C (APC) after start of COC. Evaluation of thrombin generation in women starting treatment with COC has, to our knowledge, only been investigated thrice before, by Zia et al. [29] in 2015 performed with the CAT method in the presence of 5 pM Tissue Factor (PPP Reagent), by Westhoff et al. [24] using 10 pM Tissue Factor, and by Morimont et al. [36] using ST Thromboscreen on the CAT method. Zia et al. [29] showed a statistically significant increase in ETP and peak values, as well as a decrease in lag time, both without and with addition of thrombomodulin, but the differences were considerably smaller than the present ones. Thus, the results from Zia et al. [29] showed similar, albeit lower thrombin generation than our study, indicating a higher TG response of COC when assessed on ST Genesia, using the reagent ST Thromboscreen. Morimont et al. [36] investigated thrombin generation in COC users containing EE and levonorgestrel using the same reagent as us but on the CAT system and showed results comparable to our results (-TM). They also reported that use of COC containing estetrol and drospirenone, had a lower thrombin generation compared to the groups receiving EE in combination with either levonorgestrel or drospirenone. A case report by Zermatten et al. [35] described a significantly increased thrombin generation, with and without TM, in a woman with heterozygous factor V Leiden and COC. Thrombin generation was assessed on ST Genesia, and when compared to our results, the results of their study subject would be within our specified ranges.

Supporting the increase in coagulation capacity, as measured with the thrombin generation, we observed increased levels of TAT and F1 + 2 after starting COC. TAT is formed when antithrombin has bound and inactivated thrombin whereas F1 + 2 is a part of prothrombin being released when thrombin is activated. TAT has a much shorter half-life than F1 + 2, and F1 + 2 will thus more reliably show a prolonged increase of thrombin activity. In this study F1 + 2 increased more than TAT which is in accordance with a sustained increased endogenous coagulation capacity when taking COC.

We tested antithrombin activity by two different methods and found a significant reduction when using the anti-IIa-method (AT-T), whereas antithrombin activity measured by the anti-Xa-method did not change after starting COC. We have no explanation for this discrepancy. Two previous studies [37, 38] report 9–30% reduction in antithrombin activity in COC users, whereas Zia et al. [29] showed no statistically difference of antithrombin.

It is well documented that TFPI decreases under influence of COC [22, 39–41]. Compared to previous studies [39–41], we found a modest, though statistically significant, reduction of 9%; we have only measured total TFPI and free TFPI may have shown a more marked reduction.

Protein S activity was reduced by 9% after starting COC. No statistically significant changes were seen for free protein S antigen, possibly because this method is constructed to detect low values. The literature indicates a smaller reduction in protein S for 2nd generation COC than for 3rd generation COC [33, 42–45]. Zia et al. [29], who also compared protein S activity before and after start of COC, showed a 10% decrease, consistent with our results.

Our results regarding SHBG, which showed an increase close to 90%, are consistent with previous findings [46–51]. It can be considered a marker of total oestrogenicity in the body, where increased oestrogen leads to increased SHBG to bind the higher levels of oestrogen [52, 53]. The majority of the participants received 30 µg EE, but four of them received formulations with 20 µg. Although the numbers were limited, the results are in accordance with a significantly higher oestrogenicity, as well as a tendency to higher thrombogenicity in the 30 µg-group.

The results of this study show that use of COC leads to higher thrombin generation in line with other similar reports indicating a hypercoagulable state and potentially an increased risk of VTE. Previous papers have indicated an association between an increased thrombin generation and risk of thromboses [54–56]. However, it is not known whether thrombin generation can be used to determine the risk of VTE for women using COC, but it has been suggested to be a potential tool for this [57]. The present results give an indication of reference intervals for changes of thrombin generation after start of COC giving the opportunity to evaluate the levels in women suspected to be at risk of VTE. The advantage of ST Genesia, compared to other methods, is the automated platform with specialized dedicated kits, which makes the use of thrombin generation tests in routine laboratories possible.

It is a strength of the study that we have compared the same individuals before and after start of COC, and furthermore, that all thrombin generation related blood samples were drawn into Monovette tubes, to prevent activation of the contact system [58]. Limitations include the sparse study population, but this was necessitated by an unforeseen slow recruitment. The study population limitation, made between group comparisons extremely difficult and interpretations of the results should therefore be dealt with very carefully. Furthermore, TFPI measurement was only performed as total TFPI because kits for measuring the free TFPI were not available. Another limitation of this study is the omission of measuring a normalized APC sensitivity ratio, which would have given more information, but ETP inhibition ratio can be measured on ST Genesia. The day of the menstrual cycle for the first blood sample was not set to a fixed day or interval, although it was mainly sampled around the menstruation. However, the fact that each woman was her own control will minimize the importance of this limitation, and, furthermore, Tchaikovski et al. [59] showed no significant difference of thrombin generation between blood samples drawn in follicular and luteal phase.

## Conclusion

In this paired study we analyzed thrombin generation using ST Genesia. Although COC induces several changes of especially the endogenous coagulation inhibitors, the changes of these are only modest, whereas measuring the thrombin generation as performed on ST Genesia displays a considerable change. These results give an indication of a reference for the expected change of thrombin generation after starting COC.

## Electronic supplementary material

Below is the link to the electronic supplementary material.

Supplementary Material 1

## Data Availability

No datasets were generated or analysed during the current study.
