# Supplementary material for: The effect of combined oral contraceptives on thrombin generation assessed on ST Genesia– a paired clinical study
Source: Thromb J. 2025 Apr 8;23:30. doi: 10.1186/s12959-025-00713-z (PMC11978137; doi:10.1186/s12959-025-00713-z)
Supplement: Supplementary file 1 — Supplementary Material 1 [file 12959_2025_713_MOESM1_ESM.docx]

**Supplementary material**

**Supplementary tables:**

| Supplementary table 1. Results for standard analyses, n=24 | | | | | |
| --- | --- | --- | --- | --- | --- |
| Analyte, unit | **Baseline**  Median [25p-75p] | **Follow-up**  Median [25p-75p] | **Absolute intraindividual difference**  Mean ± SD | **Relative intraindividual difference** Mean ± SD (%) | **p-value** |
| Kalium, mmol/L | 3.7 [3.6 – 3.8] | 3.5 [3.4 – 3.7] | -0.1 ± 0.3 | -2.6 ± 8.2 | 0.069 |
| Sodium, mmol/L | 139 [138 – 140] | 140 [138 – 141] | 0.2 ± 2.2 | 0.2 ± 1.6 | 0.729 |
| Albumin, g/L | 39 [38 – 41] | 40 [37 – 42] | 0.04 ± 3.47 | 0.37 ± 8.47 | 0.790 |
| Carbamide, mmol/L | 4.2 [3.8 – 4.7] | 3.8 [3.4 – 4.9] | -0.06 ± 1.15 | 1.3 ± 29.7 | 0.633 |
| Creatinine, µmol/L | 61 [56 – 65] | 65 [56 – 68] | 1.2 ± 7.4 | 2.9 ± 12.6 | 0.540 |
| ALAT, U/L^*^ | 19 [13 – 30] | 17 [12 – 25] | -4.8 ± 12.9 | -9.4 ± 35.5 | 0.123 |
| LDH, U/L | 161 [147 – 171] | 156 [140 – 173] | -1.2 ± 31.1 | 1.3 ± 21.5 | 0.877 |
| Bilirubin, µmol/L^*^ | 6.0 [5.0 – 8.0] | 7.0 [5.2 – 11.8] | 1.2 ± 5.3 | 22.5 ± 64.3 | 0.157 |
| CRP, mg/L | 0.7 [0.0 – 2.2] | 1.5 [0.0 – 2.9] | 1.8 ± 7.3 | 129 ± 471 | 0.284 |
| Cholesterol, mmol/L | 4.0 [3.6 – 4.2] | 3.7 [3.3 – 4.2] | -0.11 ± 0.65 | -1.9 ± 16.1 | 0.482 |
| HDL, mmol/L | 1.2 [1.2 – 1.4] | 1.3 [1.1 – 1.4] | 0.001 ± 0.24 | 1.8 ± 19.3 | 0.898 |
| LDL, mmol/L | 2.3 [1.8 – 2.6] | 2.0 [1.7 – 2.3] | -0.2 ± 0.6 | -6 ± 27 | 0.068 |
| Triglycerides, mmol/L | 0.9 [0.7 – 1.0] | 1.1 [0.7 – 1.4] | 0.2 ± 0.4 | 25 ± 48 | 0.066 |
| Haemoglobin, mmol/L^*^ | 7.9 [7.4 – 8.2] | 8.1 [7.5 – 8.3] | 0.39 ± 1.71 | 0.8 ± 6.6 | 0.812 |
| MCV, fL^*^ | 88 [85 – 91] | 87 [84 – 90] | 0.4 ± 2.3 | 0.5 ± 2.6 | 0.332 |
| Erythrocytes, x10^12^/L^*^ | 4.4 [4.1 – 4.5] | 4.3 [4.1 – 4.5] | - 0.03 ± 0.21 | -0.6 ± 5.0 | 0.495 |
| MPV, fL^*^ | 9.8 [9.5 – 10.7] | 10.1 [9.7 – 10.7] | 0.2 ± 0.7 | 2.1 ± 6.5 | 0.101 |
| EVF^*^ | 0.38 [0.36 – 0.39] | 0.39 [0.36 – 0.40] | 0.02 ± 0.08 | -0.2 ± 4.9 | 0.812 |
| Reticulocytes, x10^9^/L^*^ | 56 [50 – 66] | 61 [51 – 71] | 3.04 ± 14.1 | 10 ± 28 | 0.290 |
| Leukocytes, x10^9^/L^*^ | 5.9 [5.5 – 7.2] | 6.4 [5.6 – 7.2] | 0.8 ± 2.9 | 16 ± 51 | 0.406 |
| Thrombocytes, x10^9^/L^*^ | 269 [229 – 318] | 280 [223 – 317] | -6.4 ± 46.1 | -1.6 ± 16.6 | 0.665 |
| IPC^*^ | 11 [8 – 14] | 10 [7 –15] | -0.3 ± 5.9 | -3.5 ± 44.5 | 0.179 |
| IPF, x10^9^/L^*^ | 0.04 [0.03 – 0.06] | 0.03 [0.02 – 0.05] | 0.003 ± 0.03 | 4.7 ± 71.7 | 0.388 |

*n=23 due to analytical error.

Supplementary table 1. ALAT = alanine transaminase. LDH = lactate dehydrogenase. CRP = c-reactive protein. HDL = high density lipoprotein. LDL = low density lipoprotein. MCV = erythrocyte volume, mean. MPV = thrombocyte volume, mean. EVF = erythrocyte volume fraction. IPC = immature platelet count. IPF = immature platelet fraction.

| Supplementary table 2. Comparison of the 20 µg and 30 µg ethinyloestradiol groups, relative difference values | | | |
| --- | --- | --- | --- |
| Analyte | **20 µg mean ± SD** | **30 µg mean ± SD** | **p-value** |
| ETP, nmol/L.min | 39.0 ± 11.1 | 47.2 ± 25.4 | 0.539 |
| ETP (+TM), nmol/L.min | 85.1 ± 25.2 | 88.7 ± 60.0 | 0.908 |
| Lag time, min | -2.6 ± 6.5 | -1.2 ± 7.8 | 0.732 |
| Peak height, nmol/L | 78.9 ± 26.0 | 89.2 ± 58.3 | 0.735 |
| Time-to-peak, min | -10.8 ± 4.2 | -10.7 ± 6.6 | 0.993 |
| TFPI, ng/mL | -12.7 ± 6.6 | -8.9 ± 11.3 | 0.526 |
| SHBG, nmol/L | 13.8 ± 22.7 | 43.1 ± 23.7 | 0.0002 |
| F1+2, pmol/L | 15.4 ± 31.7 | 38.4 ± 44.3 | 0.03 |
| TAT, µg/L | -8.3 ± 4.0 | 19.9 ± 22.8 | 0.02 |
| AT-T | -13.5 ± 7.5 | -7.6 ± 6.5 | 0.118 |
| Protein S activity | -3.3 ± 11.1 | -6.8 ± 11.8 | 0.584 |
| Free protein S | 8.2 ± 22.9 | 4.9 ± 17.8 | 0.749 |

Supplementary table 2. ETP = endogenous thrombin potential. TM = thrombomodulin. TFPI = tissue factor pathway inhibitor. SHBG = sex hormone binding globulin. F1+2 = prothrombin fragments 1+2. TAT = thrombin-antithrombin complex. AT-T = antithrombin (anti-IIa).

**Supplementary figures:**

Supplementary figure 1:


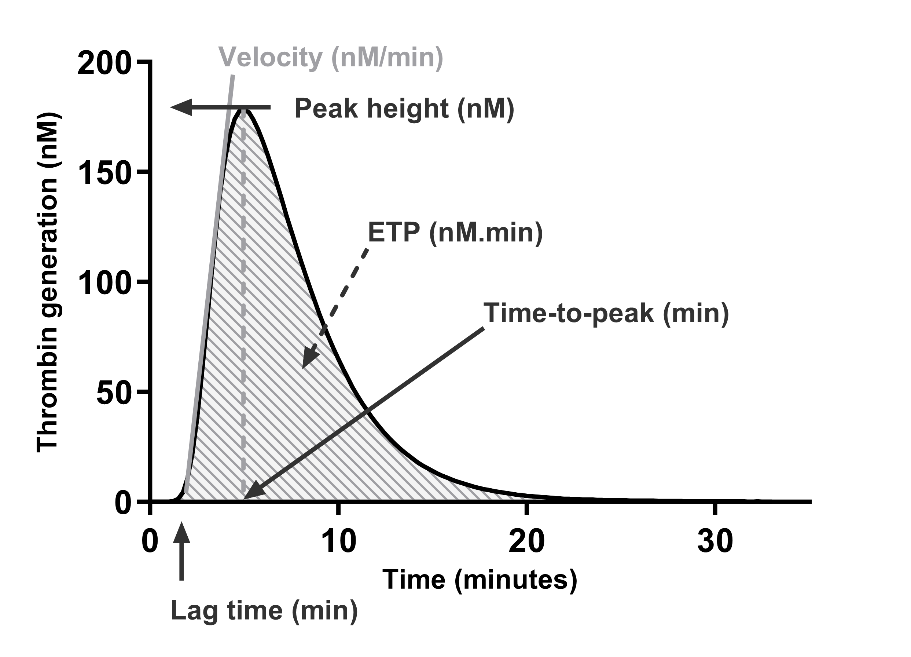


Supplementary figure 1. Thrombogram illustrating the various measurements when assessing thrombin generation. ETP = endogenous thrombin potential (area under the curve).

Supplementary figure 2:**
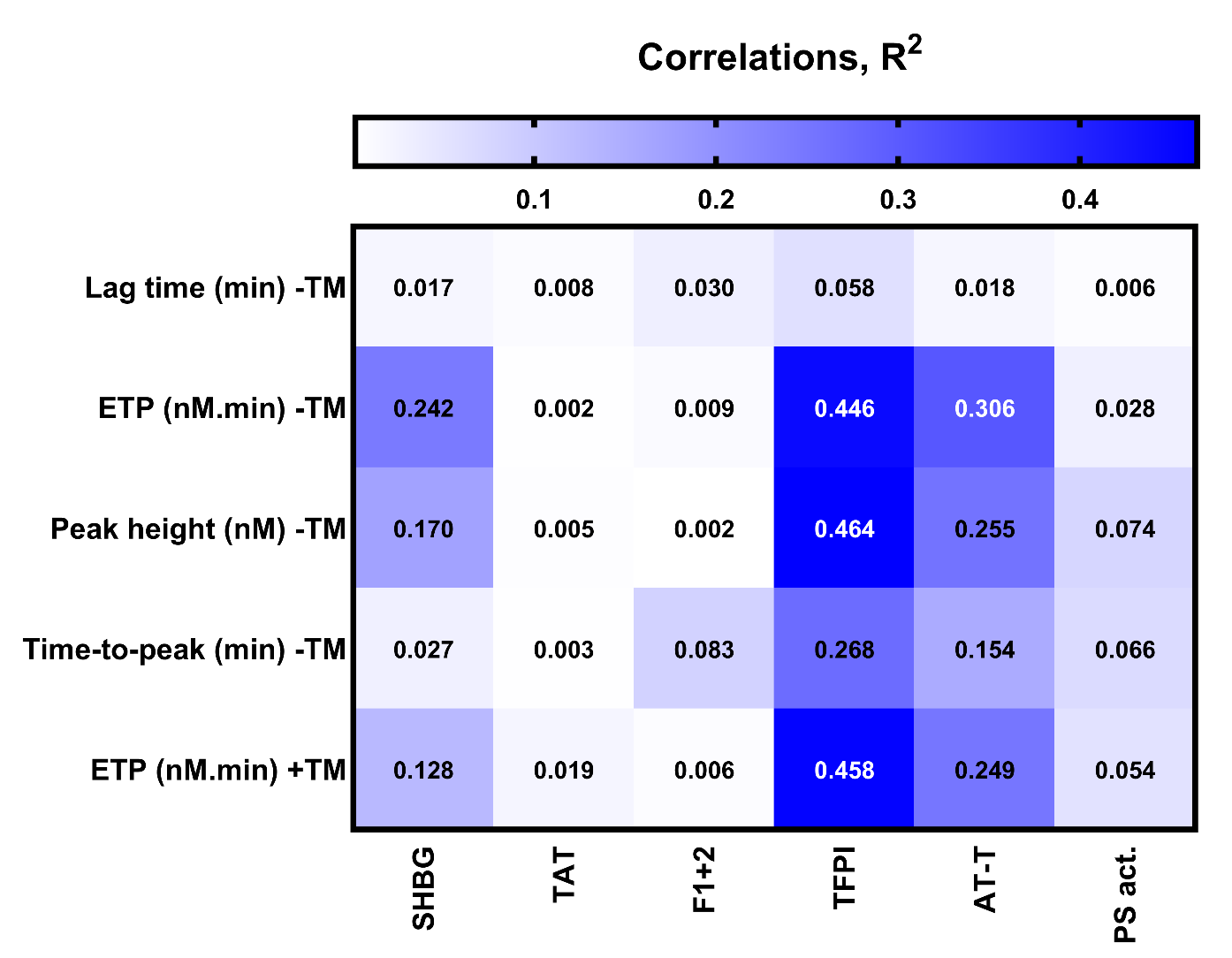
**

Supplementary figure 2. Correlations between thrombin generation parameters and changes in various analytes. ETP = endogenous thrombin potential. SHBG = sex hormone binding globulin. TAT = thrombin-antithrombin complex. F1+2 = prothrombin fragments 1+2. TFPI = tissue factor pathway inhibitor. AT-T = antithrombin (anti-IIa). PS act. = protein S activity.
